# The Ready-Reference Handbook of Diseases of the Skin

**Published:** 1901-02

**Authors:** 


THE READY-REFERENCE HANDBOOK OF DISEASES OF THE SKIN. By GEORGE THOMAS JACKSON, M. D., Professor of Dermatology, Woman's Medical College of the New York Infirmary, and in the Medical Department of the University of Vermont; Chief of Clinic and Instructor in Dermatology, College of Physicians and Surgeons, New York. New (3d) edition. In one 12mo volume of 638 pages, with 75 illustrations and a colored plate. Philadelphia and New York: Lea Brothers & Co. 1899. [Price, $2.50.]

A book whose distinguishing merits are clearly demonstrated by the fact that notwithstanding there are so many other books upon the same subject, and that the demand for the same is necessarily limited, this particular one has already passed into a third edition.

This present volume has not only been carefully revised, but numerous important additions have been made to the text on bulpiss, bunion, dermatitis from Röntgen rays, blastomycetic dermatitis, lupus pernio, pityriasis alba atrophicans and idiopathic multiple pigmented sarcoma. The additions include forty fresh pages together with six new illustrations.

Dr. Jackson still adheres to the old method of arranging diseases alphabetically, which is well enough in its way, but which lacks the advantages to be derived from systematic classification. It is likely that the author had in mind the convenience of students rather than that of professionals. At the same time the usefulness of the volume is not limited to the student class; there is, besides, much in it for both the general practitioner and the specialist. Dr. Jackson's book is remarkable for simplicity, clearness and directness, as well as for the vividness of the numerous clinical pictures. In short, it is one of the best reference books of the kind that has yet appeared—one that will be readily adopted by colleges as a standard text-book and that will be found to carry out fully its original intention. The mechanical features of the book are all that could be desired. G. W. W.

